# ‘I’m not just a vet, I’m also a human.’ A qualitative interview study on boundary management between work and private life among small animal veterinarians

**DOI:** 10.1371/journal.pone.0322938

**Published:** 2025-05-09

**Authors:** Christian Dürnberger, Svenja Springer

**Affiliations:** Department of Interdisciplinary Life Sciences, Messerli Research Institute, University of Veterinary Medicine Vienna, Vienna, Austria; University of Wisconsin-La Crosse, UNITED STATES OF AMERICA

## Abstract

**Introduction:**

Many studies of veterinarians underline the importance of work-life balance, yet our understanding of how veterinarians manage the boundary between private and professional life is still limited. In line with previous research that speaks of “boundary management” in this context, it is the overall aim of this study to investigate the conflicts and challenges veterinarians face in relation to temporal, physical and psychological boundaries between work and private life, and to explore the coping strategies they employ to navigate these challenges.

**Methods:**

The study is based on qualitative, semi-structured individual focused interviews with 20 small animal veterinarians resident in Germany (n = 8), Switzerland (n = 7) and Austria (n = 5), who specialised in the field of hospice and palliative care. A structured coding process, incorporating both deductive and inductive elements, was utilized to analyse the data through two cycles of coding, thereby identifying key themes.

**Results:**

The study identified that the veterinarians have to deal with conflicting private and professional appointments, accessibility outside of opening hours through information and communication technologies, professional concerns occupying the veterinarian’s private life and unwanted insights into the animal owners’ lives. Coping strategies show that veterinarians are willing to integrate professional aspects into their private lives and accept accompanying negative consequences because they are convinced that they are acting in the best interest of the animals. At the same time, boundary management for veterinarians means a conscious demarcation from the animal owner along temporal, physical and psychological boundaries, e.g., ensuring that the relationship remains a professional business relationship and does not become too personal. In addition, our data suggest that previous negative experiences of a lack of work-life balance led to stricter boundaries and more of a “self-care” mindset.

## Introduction

Today, people take on a variety of roles that are shaped by specific interactions, responsibilities and experiences in different societal contexts. They are, for example, a parent, an employee, a partner, a member of a tennis club, a neighbour, a consumer, a supervisor, etc. These varying roles can be assigned to two general domains, namely “work” and “private life” (or “non-work”), both driven by specific rules, thought patterns and behaviours [1].

However, the existence of these two domains urges the issue of how people can separate work and private roles, or in other words, how these roles can be reconciled in daily life. Imagine the following situations: a veterinarian is just leaving her practice to pick up her children from school when a long-term client arrives with her dog – an urgent emergency. At the weekend, the veterinarian is spending time with her family and friends, but her mobile phone keeps ringing. A pet owner sends pictures of his sick cat and wants advice. Situations like these, in which professional and private lives collide, are not uncommon in veterinarians’ daily lives, and illustrate not only the tensions between the two domains, but also the need for coping strategies. Previous research speaks of “boundary management” [2] in relation to the boundaries that individuals draw (or otherwise) in order to cope with the demands of their professional and private responsibilities, obligations and wishes [1,3].

Following Clark [1], boundary management is at least threefold: (1) Temporal boundaries refer to when tasks are completed (e.g., during business hours or after work); (2) Physical boundaries refer to the places where tasks are performed or where domain-specific behaviours are exhibited (e.g., in the office or at home); (3) Psychological boundaries are set by the individual and are expressed through thought patterns, behaviours and emotions [1].

In relation to the question of how individuals manage these boundaries, the sociologist Nippert-Eng [4] claims two types. On the one hand, “segmenters” establish a strict boundary between the domains of work and private life. For example, they do not perform any professional tasks in their free time, they might maintain two separate calendars or refrain from hanging up holiday photos in the office. On the other hand, “integrators” aim for a high integration of work and non-work; in their case, there is little distinction between what constitutes ‘home’ or ‘work’. More recently, this overlap of work and private life has also been referred to as “work-life blending” [5]. Although the boundaries can be permeable in both directions, i.e., professional aspects can intrude into private life and private issues into professional life, the corresponding empirical studies show that the former in particular represents a challenge and occurs frequently [6].

Studies show that *both* segmentation and integration strategies come with certain advantages as well as disadvantages. People who integrate their job into their private life, e.g., by being available on the phone after work or checking their e-mails in their free time, may experience flexibility and be given greater career opportunities, but the constant accessibility can lead to possible negative aspects, such as psychological stress [7], being less able to switch off from work [6] and getting less sleep [8]. Rapp et al. [9] even link boundary management to burnout, arguing that workers tend to experience exhaustion, detachment and inefficacy if their job unintentionally spills over too much into the private sphere. A strong separation of work and private life can prevent negative experiences at work from spilling over into one’s private life [10]. This separation, however, can lead to people struggling with the transition from one role to another and/or feeling that they are not fulfilling their obligations [3].

In recent years, there has been an increase in studies dedicated to boundary management in various professional fields like financial sales [11], healthcare [9], journalism [12], banking [13], nursing [14], academia [15] and the information technology sector [16]. It is astonishing that the field of veterinary medicine has so far been largely ignored in research on the subject of boundary management, especially since studies of veterinarians underline the importance of work-life balance [17–19].

Against this background, it is the overall aim of this study to gain knowledge about veterinarians’ actual boundary management by investigating how they manage the boundaries between their work and private life. Twenty semi-structured individual focused interviews were conducted with small animal veterinarians specialising in the field of hospice and palliative care, working in Austria, Germany and Switzerland. Of the 20 veterinarians, 19 were self-employed. The selected group is a relevant study population for this context for the following four reasons:

(1) Regarding veterinary medicine in general, studies show that the risk of burnout is higher in the veterinary profession than in other professional groups [20], and identify a poor work-life balance as an essential driver [19,21,22].
(2) Regarding the field of hospice and palliative care, it has been shown in human medicine that repeated confrontation with the dying process and the death of patients can be associated with psychological stress for hospice nurses [23]. Hoffmann and Dickinson [24] emphasised that veterinarians working in the field of hospice and palliative care find the mental challenge of end-of-life care particularly demanding. It can therefore be assumed that the examined field of work includes emotional cases that can potentially preoccupy healthcare professionals in their free time.
(3) Studies on other veterinary professional fields, including bovine practitioners, show that the unpredictability of veterinary working hours and on-call duty in particular lead to conflicts with private life [25]. This also applies to hospice and palliative care in small animal practice since this occupational field is also particularly affected by acute, unplannable emergencies, which potentially intensify challenges at the boundary between work and non-work.
(4) Finally, while boundary management is often strongly influenced by employers [26], self-employed individuals must set limits themselves without losing sight of their income, their work-life balance, and the interests of their clients [27,28].

In light of these four aspects, it can be expected that self-employed veterinarians working in the field of animal hospice and palliative care are confronted with challenges in the area of boundary management and must develop coping strategies to deal with them. This study assumes that veterinarians – self-employed veterinarians in particular – tend towards the “integrator” type. This assumption appears plausible considering that the study posits a high degree of professional identification among veterinarians. Previous research in other occupational fields has demonstrated that a strong identification with one’s professional role often correlates with an increased willingness to integrate that role into one’s private life [3,15].

The present study is part of a larger project that focuses on veterinarians’ work experiences in the field of hospice and palliative care in small animal practice. In the first step, Springer et al. [29] reveal relevant insights to enhance our understanding of the sources of veterinarians’ motivation to build competencies in the field of animal hospice and palliative care, as well as the extent to which relationships, communication, time, and infrastructure play a role in the care of chronically and/or terminally ill patients. The second step focuses on the following two research questions: (1) What conflicts and challenges do small animal veterinarians in the field of hospice and palliative care face in relation to (a) temporal, (b) physical and (c) psychological boundaries between work and private life? (2) What coping strategies do they use to deal with these conflicts and challenges?

## Materials and methods

The study is based on individual focused interviews with 20 small animal veterinarians resident in Germany (n = 8), Switzerland (n = 7) and Austria (n = 5), who specialised in the field of hospice and palliative care (as certified by the International Association for Animal Hospice and Palliative Care (IAAHPC)) and/or explicitly mentioned and advertised on their practice website). Of the 20 interviewees, 19 were self-employed, while 1 was employed, having previously been self-employed for many years. The group comprised 18 women and 2 men. Detailed information on country, gender, working relationship (self-employed or employed), professional experience in years, duration of the interview and the interviewers can be found in the overview table as part of the S1 File.

### Recruitment process and study participants

To initiate the recruitment process for veterinarians participating in our study, we conducted an online search for veterinarians, practices and/or clinics in the small animal sector, targeting specific regions. For Austrian participants, we utilized the platform Herold.at on June 16, 2022. Next, we focused on German participants and performed our search using 11880.com from June 26 to June 29, 2022. Finally, for Swiss participants, we turned to local.ch, conducting our search on July 14, 2022. Additionally, to ensure a comprehensive outreach, we performed an extra online search using Google.at on August 6, 2022. Using relevant search terms, including “veterinarian”, “palliative”, “hospice” and “small animal practice”, 37 additional veterinarians, practices and/or clinics in the small animal sector (Germany n = 28; Switzerland n = 2; Austria n = 7) were identified that were not displayed in the classified directories. Through these online searches, we aimed to compile a diverse and qualified pool of veterinarians for our study.

In total, 3,007 websites were reviewed. Results of the online search showed that only 52 websites (2.4%) mentioned the service of hospice and/or palliative care. From these 2.4%, potential study participants were selected based on the following considerations: Various aspects (e.g., diversity in the type of practice and specialisation, years of work experience, degree of urbanisation and work place) were considered in order to obtain a broad spectrum of experiences and thereby increase the chance of transferability. In total, there were three recruitment phases. On December 7, 2022, the first recruitment phase started by contacting nine selected veterinarians in Germany, five in Switzerland, and six in Austria. After this first phase, 16 vets (seven in Germany, four in Switzerland, and five in Austria) confirmed their participation. In the second recruiting phase, on February 14, 2023, three German vets were contacted and one vet from Switzerland. In this phase, one more veterinarian in Switzerland agreed to participate. In the third phase, on March 10, 2023, two veterinarians in Switzerland were contacted, with one veterinarian agreeing to participate in the study on March 17, 2023, so that the recruitment process was finished.

In January 2023, two pilot interviews were conducted with veterinarians specialising in palliative and hospice care, aligning with our target population. The primary aim of these pilot interviews was to assess the clarity and comprehensibility of the interview questions and to ensure that no important topic was missing. Based on their feedback, we confirmed that the questions were well understood and made minor refinements to improve wording where necessary. No formal validation process (e.g., external expert review or structured bias assessment) was conducted. As one of the two pilot interviews ran smoothly, we decided to use the corresponding data from this interview in the final sample of the study. Participants were offered €50 compensation for participating in the interview study. Following the ethical approval process at the University of Veterinary Medicine, Vienna, the study was submitted for ethical approval to the Ethics Committee of the Medical University of Vienna before the start of the recruitment process. After reviewing the study, the requirement for further ethical approval was waived by the committee.

During the recruitment process, it was difficult to find male veterinarians, not least because there are more female than male veterinarians working in this field. We therefore decided to accept the low number of participants in this sampling stratum. Before the study, all participants were provided with information about the type of data that would be recorded in the study and how it would be handled, that participation was voluntary and that they were free to withdraw their consent at any time during or after the study. All participants provided written consent and no participants withdrew their consent during or after the study.

### Data collection procedure and structure of the interviews

All 20 interviews were conducted in German and online using a web conference tool (Cisco Webex) through which they were also video recorded. The duration of interviews ranged from 48 to 100 minutes, with an average duration of 73 minutes. All interviews followed a semi-structured interview guide. This consisted of four parts and six themes (S2 File). The development of the interview guide was grounded in the theoretical framework proposed by Springer and Axiak Flammer [30], which suggests four key aspects particularly relevant to veterinary hospice and palliative care: relationships, time, communication, and infrastructure. Questions were formulated to explore these domains.

Individual questions were formulated during the interviews slightly differently and changed and adapted to suit each participant and his or her working background. The senior author of this paper (S.S.) conducted 18 interviews, while the first author (C.D.) conducted two interviews.

### Data analysis

Recordings were transcribed verbatim and coded using the MAXQDA 2020 (version 20.4.2) software program (Berlin, Germany). Following the template organising style [31], categories and codes were created and formulated based on the key aspects of the interview guide, the research questions and the hypotheses of the project. A first and second cycle of coding was conducted to analyse the data gained from the 20 interviews [32]. Using a deductive approach, the overall aim of the first cycle of coding was to summarise segments of data and categorise similar data units [32]. The deductive approach in the first coding cycle was guided by the conceptual framework introduced by Springer and Axiak Flammer [30], which identifies key domains in veterinary hospice care. These domains provided the initial categories for coding, ensuring alignment with the pre-established theoretical framework. The initial code list for the first cycle of coding contained nine categories with a total of 40 codes. During the first coding cycle of two interviews (conducted by S.S. and C.D.), this initial list was adjusted to include two new codes and to adjust codes to make them more applicable for the analyses. These alterations chiefly followed an inductive approach based on obtained data. The final code list includes nine categories with 42 codes (see S3 File). Categories, and in particular codes, were continually discussed by the project team (S.S. and C.D.) to ensure the relevance and use of codes, especially during the first cycle of coding. During a second cycle of coding, the initial results were grouped into smaller categories and clusters to gain more meaningful units for subsequent content analysis [32]. The interviews were conducted and transcribed in German, and our categorisation and coding framework was ultimately developed in English. Throughout this translation process, we remained cognizant of potential conceptual and cultural differences that might arise. To mitigate any issues, we employed a collaborative approach, where both authors engaged in discussions regarding the nuances of the language and meaning. Although we encountered minor linguistic challenges with certain quotes, we ensured that their essence was preserved by carefully selecting translations that accurately reflected the original context. Moreover, we acknowledged specific cultural constructs intrinsic to the German language that may not have direct English equivalents. In reporting our findings, we provided additional explanations for these concepts to facilitate reader understanding, thereby enhancing the transparency and rigor of our qualitative analysis. This thorough approach not only helped reduce the possibility of errors in translation but also addressed potential biases associated with language differences, ultimately contributing to the fidelity of our research findings.

This interview study is a qualitative social science study with an explorative character. The aim was not to make statistical generalisations about a background population, but rather to explore veterinarians’ boundary management and coping strategies.

## Results

### Conflicts and challenges

**Fig 1:**
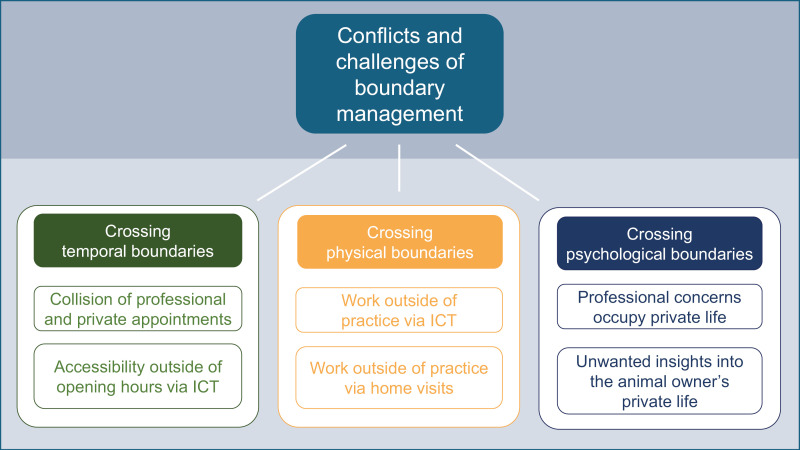
Overview of identified conflicts and challenges with respect to the three types of professional boundaries.

The study identified several conflicts and challenges with respect to the three types of boundaries (see Fig 1): Turning to the temporal boundaries, the collision of professional and private appointments, as well as accessibility outside of opening hours via information and communication technology (ICT) can be challenging. Furthermore, our findings indicate that work outside of the practice via ICT and home visits brings with it a crossing of physical boundaries. Finally, in terms of psychological boundaries, our results show that professional issues can take over veterinarians’ private lives and that veterinarians can gain unwanted insights into the pet owners’ private lives.

### Crossing temporal boundaries

(a) **Collision of professional and private appointments**

Sudden professional obligations can collide with the veterinarian’s private life. Corresponding conflicts and challenges primarily affect family life. An exemplary quote reads:

*“When the children were younger, this was still pretty intense. Because if you’re trying to drive to the movies with two kids and then someone calls [and says] ‘my dog has this and that’, and you say, ‘no, you can all get out of the car again.’ Not good. Not a good story. In other words, there have been many lessons in how to solve these things without hurting others.” (Interview (Int.) 4, Position (Pos.) 155)*

One interviewee described the possibility of an emergency occurring as follows:

*“And of course, that’s difficult in the holidays, because you want to spend a few days with your children on vacation and you know that the dog [i.e., a patient animal; C.D.] could be very unwell at any time. And then it’s always a bit of a lottery as to whether it will go well, whether it will fit into your plans or not.” (Int. 5, Pos. 47)*

Professional appointments invading the “private” domain can also happen in the case of illness. The following quote describes such a situation, which the veterinarian perceived as a violation of boundaries by the animal owner:

*“[...] [W]hen I was ill during the week [...], I also had people calling me to ask if I could come now. And I didn’t have a voice, but I said on the phone: ‘I really can’t come now.’ ‘Yeah, I can hear that you’re ill, but can you come?’ It was like, ‘I don’t care how you are. I want you to come.’ Right? Where I think to myself, okay, hmm, good. That’s difficult.” (Int. 15, Pos. 56)*

The quote shows by way of example that corresponding challenges are often described as conflicts with the animal owner. For example, one veterinarian said that some owners *“have no empathy for me (...) and for my boundaries. No understanding of the fact that I’m not just a vet, but also a human and a mother and everything...”* *(Int. 15, Pos. 50)*. Regarding the collision between professional and family life, one interviewee used verbs such as “buffering” and “juggling”: “*I buffer all my life at work and with the children [...]” (Int. 17, Pos. 65)* and *“[…] I’m always juggling children and work anyway [...]” (Int. 17, Pos. 151).*

(b) **Accessibility outside of opening hours via ICT**

The veterinarians surveyed tended to be available via ICT outside of practice opening hours – at least for certain animal owners in certain situations, in particular for the acute phase at the end of an animal’s life. For example, one veterinarian stated:

*“Then I don’t just finish at eight o’clock on Friday evening, I’m simply available then. And especially when it gets to the dying phase or when you’re expecting it, I’m also there at the weekend and available for them [the patient and owners; C.D.].” (Int. 12, Pos. 55)*

Other veterinarians reported a similar approach:

*“[...] then my mobile phone is not set to silent, day or night, [...] the person knows that they can always call me.” (Int. 15, Pos. 86.)*

*“[...] when it really comes to end-of-life care, for me that means I’m there. I’m really there. So I also have my mobile phone, I keep checking [...]” (Int. 16, Pos. 26)*

One study participant also explicitly mentioned the fact that she uses one mobile phone for both domains of her life – work and private life:

*“No, I don’t have a special business mobile phone, it’s also my private one, and that’s […] sometimes a bit difficult, to distance yourself. But if I know that an animal is really in its last phase, then I can also be reached at the weekend.” (Int. 8, Pos. 83)*

In response to the question “are you available 24 hours a day?”, one veterinarian replied: *“At the moment, more or less yes [...]” (Int. 20, Pos. 228).* The disadvantages of this constant availability were discussed by many interviewees. A typical example is the following story about an animal owner: *“[…] the first message came at six in the morning and the last at eleven in the evening. And that’s every day. And that’s when I realised I was reaching the limit of my patience.” (Int. 15, Pos. 50).* The veterinarian complained in general about animal owners *“[…] who have the expectation that I’m available 24/7”. (Int. 15, Pos. 50).*

### Crossing physical boundaries

(a) **Work outside of practice via ICT**

It became clear in the previous point that veterinarians say they work not only in the practice, but sometimes also at home, for example when they give advice to pet owners via text messages at weekends or after work. Modern ICT plays an essential role here. A typical statement is as follows:

*“So the channels are actually e-mail, SMS, WhatsApp. [...] With WhatsApp, they can also send me videos if they are unsure about the current situation, voice messages and so on, thats implifies a lot of things. [...] [S]o when videos come in, it’s usually because the owner isn’t sure how [the] pet is doing at the moment.” (Int. 15, Pos. 76)*

(b) **Work outside of practice via home visits**

Some of the interviewees also explicitly offer home visits, as they believe these improve patient care, particularly in the context of hospice and palliative care. One exemplary statement reads as follows: *“And when it comes to euthanasia or end-of-life care, the animals are usually very sick and old (…), where it is simply more difficult to transport them.” (Int. 8, Pos. 117).* When the veterinarians talked about these home visits, they made comparisons with the “practice/clinic” setting and described the latter as a place where it is easier to distance themselves emotionally from the animal owners and their situation:

*“It’s easier to distance myself emotionally and protect myself in a clinic setting than when I’m at people’s homes […]” (Int. 15, Pos. 40)*

*“And a certain advantage for me in practice is that it’s easier to keep my distance from the client because the framework is simply different. That also has advantages for me in that it’s easier to keep a bit of distance.” (Int. 20, Pos. 189)*

*“In the practice, it’s much easier to remain objective and professional without getting a lump in your throat, isn’t it?” (Int. 18, Pos. 148)*

### Crossing psychological boundaries

(a) **Professional concerns occupy private life**

In some cases, veterinarians have to think about professional concerns even after work. One veterinarian described this situation as follows: *“And these are also things like that, [...] you don’t just surrender them at the door and go home.” (Int. 2, Pos. 109).* Another interviewee stated: *“[...] there’s nothing worse than lying in bed at night and feeling like, oh, that wasn’t good, [the owners] have all experienced a real drama and that’s bad.” (Int. 12, Pos. 79).* According to one interviewee, these concerns can even affect their sleep: *“So, I know friends from my studies; they really chew on it. Or they have nightmares…” (Int. 11, Pos. 109).*

The professional concerns that prevent veterinarians “switching off” mentally after work do not relate to the health status of the patient, but rather to the situation of the animal owner. For example, euthanasia of an animal is not perceived as stressful per se, yet what can still cause stress after work is the pet owner’s grief and emotional outbursts. One veterinarian said the following about the euthanasia of a dog: *“[T]hen suddenly the […] man [...] sits there crying”* and then *“you take these worries and this grief with you to a certain degree [...]” (Int. 5, Pos. 69)*.

(b) **Unwanted insights into the animal owner’s private life**

Another challenge described by many veterinarians is a situation where the animal owner’s private life spills over into the veterinarian’s professional life. In such situations, veterinarians are confronted with a lot of personal information from the owner. In general, almost all respondents stated that they knew a lot about the lives of animal owners: *“We know everything about our owners.” (Int. 13, Pos. 87).* In their conversations with the veterinarian, pet owners not only talk about their animals, but also about important topics such as *“[the] death of the husband or children, or […] cancer.” (Int. 2, Pos. 73).*

This particular type of boundary management challenge, e.g., finding out things about the client that the veterinarian might prefer not to know, is particularly evident during home visits. Typical quotes are as follows:

*“[...] I don’t want to see how people live, especially older people, it’s so depressing in their big empty apartments with the old furniture, where they’re alone with domestic help and an old cat that you then kill, so to speak. I just don’t want to see all that, because I often find it very depressing, because it’s not necessarily the lovely, beautiful home where the golden retriever is lying on the white couch [...]” (Int. 1, Pos. 213)*

*“Yes, really sad things in some cases, where you simply see that they live at a subsistence level, there’s nothing in the fridge. There’s really nothing in the fridge and then they have a sick animal. And what are you going to do? [...] Or […] an old grandmother in her 80s who has a cat that’s 20. She’s only still on this planet because this cat is still here. But the cat is terribly ill, and nobody looks after granny. [...] [T]he water is turned off and the electricity has already been cut off and it’s really just terribly sad, of course you take that home with you and think to yourself, for God’s sake, how bad.” (Int. 12, Pos. 119)*

Describing home visits in general, one veterinarian said there was a *“danger that it becomes very personal [...]” (Int. 20, Pos. 192)* between the veterinarian and the animal owner.

### Coping strategies

**Fig 2:**
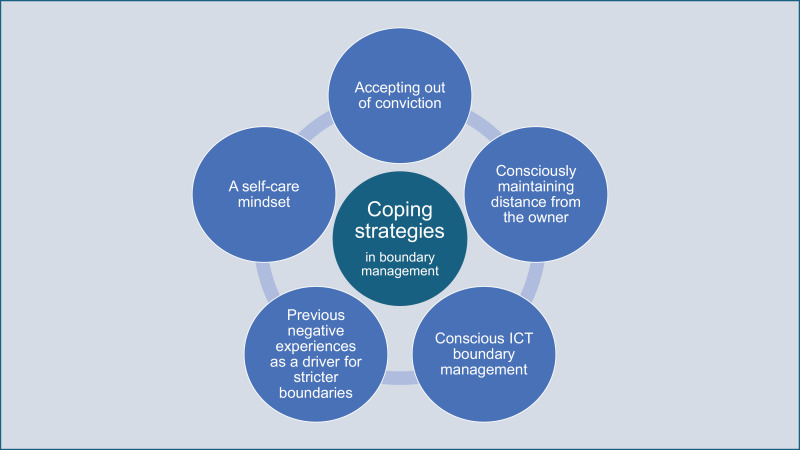
Overview of identified coping strategies in the context of boundary management.

Our results show that veterinarians try to cope with boundary management challenges by accepting out of conviction, consciously maintaining distance from the animal owner, conscious ICT boundary management and/or a self-care mindset. Furthermore, data suggest that previous negative experiences of work-life balance constitute a driver for stricter boundaries between work and private life (see Fig 2).

### (a) Accepting out of conviction

Results show that many veterinarians just accept that their professional life can spill over into their private life. This acceptance is based on the conviction that they are acting in the best interests of their patients as well as providing a necessary service for animal owners:

*“And I also have to say that if I’ve been with an animal like this for its whole life, then I simply don’t want it to spend its last day in a clinic or emergency practice […], somewhere where this animal is not known, where they don’t know how to approach it or how it would like to be addressed. [...] That’s why I’m willing to do this [...], and then I also give out the telephone number where I can be reached.” (Int. 6, Pos. 15)*

*“And that’s terrible for me... even for me, I have to tell you, if I knew that the animal needed me,and I wasn’t available. So I just feel the need to help them.” (Int. 10, Pos. 55)*

### (b) Consciously maintaining distance from the animal owner

The majority of veterinarians stated that they consciously maintain a certain distance from the animal owners. For example, one veterinarian said the following:

*“You mustn’t confuse things. They [the animal owners; C.D.] are [...] not friends. We are close to each other. But we’re not friends, there’s a difference.” (Int. 4, Pos. 121)*

In this context, one veterinarian said that she *“often has to slow down animal owners” (Int. 13, Pos. 89)* so that the relationship does not become too personal. One strategy often mentioned for maintaining distance is not to be on first-name terms:

*“So I keep my distance for my own well-being as best I can. But I don’t try to be cold in any way, I simply stay correct. I use “Sie” [polite form of address; C.D.], not “Du” [indicates first-name terms; C.D.].” (Int. 1, Pos. 137)*

*“Sie” [polite form of address; C.D.] is such a boundary for me, because as soon as you use “Du” [indicates first-name terms; C.D.], it just becomes much more personal, I think.” (Int. 15, Pos. 80)*

Maintaining distance can also be achieved by the veterinarians deliberately revealing little about themselves: *“[...] Even if the animal owner shares a lot with me, I don’t necessarily have to do the same. That’s my border.” (Int. 15, Pos. 80).* Statements like the following, where the interviewee finds it easy to maintain the described distance from the animal owners, are the absolute exception: *“[...] I have to be honest, I am very good at setting boundaries with people, but not so well with animals.” (Int. 13, Pos. 70).*

### (c) Conscious ICT boundary management

As shown above, the use of ICT plays a relevant role in veterinarians’ boundary management. The study therefore not only identifies that the veterinarians see the need to be available to certain animal owners in certain situations at any time, but it also shows a conscious demarcation from the animal owners. For example, several veterinarians reported that they cannot be reached by phone at weekends, but by text message. One veterinarian justifies such boundaries as follows: *“[...] I am not the property of the owners, I am not the property of society; just because I am a veterinarian does not mean I have to be available around the clock [...]” (Int. 5, Pos. 127)*

### (d) A self-care mindset

Veterinarians not only take conscious action in relation to animal owners, such as not being on first-name terms, but also emphasise that a certain mindset is needed to master the corresponding boundary management challenges. This mindset can be described as “self-care.” A veterinarian with 30 years of professional experience stated the following:

*“Then of course you also need a certain level of self-care, to say, I accompany these animals, I accompany the people who belong to them, which is sometimes even more difficult, without overexploiting my emotions. So I have to be able to bear it. I have to be able to carry it on my shoulders without harming myself.” (Int. 6, Pos. 29)*

This self-care mindset focuses on the veterinarian’s own load limits:

*“But you have to look a little bit at where my limit is, what do I want to bear? [...] I noticed for myself [...] that I can only handle a certain number of patients per day depending on the severity [of their condition; C.D.]. I don’t mean that in a bad way, but at a certain point I notice that I am so emotionally exhausted that if I had someone [another patient; C.D.] now, I wouldn’t be able to go with them anymore. Or I wouldn’t be able to listen to them anymore because it’s just too much for me. And I think that’s a very individual limit in terms of resilience, and you have to find that out for yourself first […].” (Int. 15, Pos. 42)*

Accordingly, the respondents hoped that the next generation of veterinarians would be able to take better care of themselves:

*“I would like for this generation that they don’t have to work as hard as our generation, that they find a little balance in their private lives.” (Int. 14, Pos. 154)*

*“So for the future generation, I would like for [...] them to fully internalise that they are veterinarians and humans, that they do not identify [solely; C.D.] with their job. That it is a part of them but does not define their personality. [...] If I only identify with my job, I fall apart.” (Int. 15, Pos. 164)*

The same veterinarian therefore insisted on: *“Self-care, self-care, self-care.” (Int. 15, Pos. 166).* However, there are also rare dissenting voices who accuse the younger generation of not identifying enough with their profession and paying too much attention to their work-life balance. For example, a male veterinarian wished the following: *“I hope that the next generation will see their profession as a calling again.” (Int. 18, Pos. 174).*

### (e) Previous negative experiences of work-life balance as a driver for stricter boundaries

Some veterinarians tend to be more careful about drawing a strict boundary between work and non-work by recognising that it is important that their job does not dominate their private life as much as it used to. The participants quoted below are all female, self-employed and have many years of professional experience behind them.

Respondents talk about previous negative experiences with boundary management from which they have learned – and they have learned the hard way. A veterinarian with over 35 years of professional experience, for example, described how she is now much better at *“sensing who is draining me” (Int. 14, Pos. 114)*, i.e., which animal owners are costing her too much energy. The interviewee went on to describe how important it is…

*“[...] to really say, I’m making a cut here, no, this is the end. And I’m not going to let that get to me anymore and I’m not going to take it home with me. That’s what I did when I was young, I took every problem that someone had home with me, that I tried to help them at that point and you can really exhaust yourself in doing so and I think that’s what a lot of veterinarians do […]” (Int. 14, Pos. 114)*

A veterinarian with 16 years of work experience talked about her burnout, which she closely associated with boundary management conflicts:

*“[...] due to exhaustion [...] that I had at the beginning of the year, I’m trying to organise things better, to say I’ll do one or two [patients; C.D.], then I’ll take a break [...], and then I’ll be done, so that I still have time to do my office work, which in previous years I did at ten o’clock at night. But that’s a learning process for me because I’ve never been self-employed. I didn’t know how it would work for me without [...] my other life falling by the wayside.” (Int. 15, Pos. 142)*

Another participant with almost 30 years of work experience explained the following about her experiences of burnout:

*“I’ve had it twice. The first time I had total burnout, where I really noticed that I was lying in bed in the morning, the phone was ringing and I started to cry. Where I noticed that I couldn’t answer the phone anymore, I just couldn’t manage it anymore. I would like to help but I have no idea how, everything is just [...] I can’t do it anymore. And then I went to the doctor and thought, well, let’s see, she’ll probably send me home straight away and laugh at me. And after five minutes she gave me a sick note and said: ‘You have burnout, you’re on sick leave now. For now, you are no longer working at all.’ Then I looked at her in surprise. […] I was on 100% sick leave for three months, then slowly worked my way back up to 100% over the course of a year. It was only after I was on sick leave that I really noticed how completely worn out I was. It was a very intense experience. […] And then I caught Corona, and then I was in a burnout situation again, where I realised that I just needed more time off to recover. The 10 days that I was locked up for weren’t enough to get fit again.” (Int. 20, Pos. 221,223)*

The veterinarian quoted above also had an assumption as to why she slipped into burnout. She thereby implicitly addresses boundary management by arguing:

*“Because the temptation is great when you really enjoy doing something – and I think most veterinarians do it with passion and enthusiasm – that you simply overdo it. You want to be there for the client and their animals 24 hours a day, and that’s not possible.” (Int. 20, Pos. 227)*

## Discussion

Dealing with the conflicting demands of work and private life is particularly important for challenging professions such as the veterinary profession. A lack of work-life balance is one of the most commonly reported stressors by veterinarians [18,19] and, alongside workload, it is the strongest predictor of exhaustion in young veterinary medicine professionals [17]. A good work-life balance is recognised as the most important predictor of high levels of well-being, low burnout and good mental health among veterinarians [21]. The North American Veterinary Medical Education Consortium (NAVMEC) even lists being able to balance work and life as one of the core competencies required of graduating veterinarians [33]. Against this background, it was the overall aim of the study to provide empirical insights into veterinarians’ boundary management, and identify challenges and conflicts along temporal, physical and psychological boundaries, as well as corresponding coping strategies.

### Self-employed veterinarians are “integrators” out of conviction

The assumption that self-employed veterinarians tend towards the “integrator” type in the sense of Nippert-Eng [4], i.e., that they are willing to integrate their profession into their private lives, is largely confirmed based on our findings. This tendency reflects a conscious decision by veterinarians who are willing to make certain sacrifices in their private lives, such as being available outside of practice hours in order to provide the best possible care for their patients and the caregivers. The study refers to this as acceptance of boundary conflicts out of conviction. This result is in line with previous research, which indicates that a high level of identification with the professional role goes hand in hand with a greater willingness to integrate the professional role into one’s private life [3,15]. Economic reasons for being available around the clock, which we might have expected from self-employed individuals, were not mentioned at all in the veterinarians’ responses.

### Juggling care work at home and at work—The roles of mother and veterinarian

The willingness to integrate professional aspects into private life is associated with specific challenges. Thereby, a classic boundary conflict is the collision of professional obligations and family life. Kogan and Rishniw [34] found that the desire for more free time and more time for family and/or friends was the most common reason given by veterinarians working in clinical practice when planning to reduce their working hours or even stop working as a veterinarian altogether.

As this interview study almost exclusively surveyed female veterinarians, the role of mother came up repeatedly. One veterinarian used the word “juggling” in this context – a word that is quite common to describe conflicts between private life with children and professional life [35].

In general, recent research suggests that gender can play a decisive role in boundary management, indicating that women tend to encounter more negative consequences when work obligations intrude into their non-work life, whereas men are more prone to negative outcomes when their non-work responsibilities interfere with work [36].

According to Myrie and Daly [35], self-employed mothers were more likely to feel guilty if they spent time at work instead of with their children. Research indicates that female veterinarians in particular are affected by burnout [37] and are more likely to report stress [38]. Family obligations could play a role here, however studies [39] show that female veterinarians with children do not have higher stress levels than female veterinarians without children, concluding that work-family conflicts may not be a major source of stress for female veterinarians.

Based on our study sample, comparisons between male and female veterinarians are limited. Future research should increasingly focus on gender aspects in veterinarians’ boundary management by addressing the question of whether and to what extent men experience such conflicts differently compared to their female colleagues.

Generally, the present study did not examine the act of role transition itself. Ashforth et al. [3] speak of “micro-transitions”, e.g., the transition from the role of mother to the role of veterinarian and back, and they point out that people often – consciously or unconsciously – develop certain routines to switch between “work” and “private life”. Since such routines can also be understood as an essential part of boundary work, an interesting research task would be to identify these in the context of veterinary medicine.

### The underestimated importance of ICT

The particular importance of ICT became clear both in terms of challenges and coping strategies. This is in line with previous studies focusing on other occupational fields that underline the relevance of dealing with ICT when it comes to boundary work. For example, Barber and Jenkins [8] identified that setting stricter boundaries on work-related ICT at home positively impacts mental health by improving sleep and mental detachment from work.

Our findings suggest that in certain situations veterinarians can perceive the use of ICT as stressful, e.g., when animal owners use this means of contact excessively outside of opening hours. At the same time, it has to be noted that veterinarians consciously offer this form of communication and also describe it as a helpful tool, e.g., videos of animals can convey a better impression than descriptions by the owner. Again, this willingness to offer these forms of communication is in line with general findings that employees with higher job involvement were more likely to use communication technologies like mail or messenger outside of working hours [6,15]. However, from the point of view of the veterinarians interviewed, using these tools necessitates drawing a line. For example, the veterinarians disclosed that they carefully consider when and to whom they give their mobile phone number, or that they clearly communicate to their clients that they cannot be reached by phone at weekends, only by text messages.

Despite a growing interest in exploring the importance of communication in veterinary medicine, which argues that communication skills are a central competence in the veterinarian-client relationship [40], the essential role of ICT has so far been almost completely neglected in this context. Our data reveal that veterinarians consciously offer their availability to pet owners in certain situations, but at the same time use strategies of demarcation. Future veterinarians should be prepared for the fact that ICT boundary management will be an important aspect of their work. Further, we can expect that the use of ICT will steadily increase, so it is also necessary to discuss the question to what extent and how veterinarians charge for such services via ICT.

### Unwanted insights and the practice as a “safe harbour”

The study identified a challenge that can be described as a special case of boundary management, namely situations in which the pet owner’s private life unintentionally spills over into the veterinarian’s professional life, e.g., the veterinarian is confronted with too much personal information from the pet owner. This happens in particular during home visits. This concretises and complements previous research findings that describe issues with clients as a central challenge of the veterinary profession [41]. For instance, Andela [42] found that clients who are aggressive, cannot pay, expect too much or do not treat their animals well are particularly challenging for veterinarians. Our findings go further by not only indicating that clients’ difficult circumstances such as poverty or loneliness are challenging, but also that it makes a difference whether the veterinarians only learn about these circumstances through conversations in the veterinary practice or actually experience and sense them during a home visit. In turn, our study suggests understanding the practice as a kind of “safe harbour” of professionalism that makes it much easier for the veterinarian to remain in their professional role and maintain the necessary distance from the pet owner.

### Negative work-life balance experiences lead to stricter boundaries

Previous research has identified long working hours as a factor that can contribute to stress, anxiety and depression in veterinarians [25,39,43]. Nolen [44] found that the primary reasons for early retirement among veterinarians are related to work-life balance and mental health. Montoya et al. [45] identified similar reasons why veterinarians leave clinical practice. Veterinarians who report meeting up with friends, spending time with family or exercising frequently experience much less burnout than those who seldom do those activities [21]. Our data suggest that it is primarily past negative experiences with a lack of work-life balance that have led to stricter boundaries and a greater focus on a self-care mindset. Veterinarians stated that they had learned this the hard way and now place more emphasis on ensuring that work does not encroach too much on their personal lives.

### Demarcation from the pet owner as a core task

Veterinary boundary management means one thing above all: demarcation from the animal owner. This is evident in almost all identified conflicts, challenges and strategies. In this context, the pet owner can be described as an “intruder” who invades the domain of the veterinarian’s private life. This intrusion can be intentional, e.g., if a pet owner expects a veterinarian to be available around the clock or even when they are ill. However, it can also happen unintentionally, e.g., due to the emotionality of the situation surrounding a seriously ill animal. For instance, our data show that when professional matters occupy a veterinarian’s thoughts after work, it is more often about the pet owner’s difficult situation than the animal.

Generally, situations at the end of an animal’s life can be particularly emotional for pet owners and veterinarians [46]. While it is crucial for veterinarians to show empathy in these moments, they must also maintain a degree of professional distance. More than that, the professional role of the veterinarian seems to be largely characterised through the act of demarcation from the animal owner’s situation. This dynamic is evident both mentally and linguistically. For instance, the German language enables social distancing through the use of the formal ‘Sie’ instead of the informal ‘Du’. It would be interesting to examine the extent to which similar mechanisms are present in other languages, particularly within the context of the veterinarian-client relationship.

In other words, while it is accurate to say that client relationships are a key challenge of the profession [25], veterinarians try to shape this relationship not only through empathy and transparent communication, but also through a conscious demarcation from the animal owner along temporal, physical and psychological boundaries.

### Limitations

The interview study comprising 20 individual focused interviews must be understood as explorative in nature, without claims of statistical generalisability or representativity of results. With their explicit specialisation in palliative and hospice medicine, the respondents form a very specific group of veterinarians, which may lead to bias in the findings. Furthermore, it was difficult to recruit male veterinarians in this context. The fact that the study was carried out in German-speaking countries may mean that some results will be less relevant in other countries. Despite careful translation of the literal quotations, it is possible that certain nuances of the original statements have been lost in English.

In recognizing the limitations of this study, it is further essential to address potential sources of bias: During data collection, participant selection may have introduced bias, as individuals who agreed to participate could possess unique characteristics or experiences that do not represent the broader population. The theoretical and conceptual framework utilized in this study appears to be a plausible choice; however, it may have inadvertently led to the omission of other relevant factors that were not explicitly mentioned or targeted in the interview guide. This oversight could further impact the richness and comprehensiveness of the data. The interviews were conducted by researchers with differing disciplinary backgrounds and gender, which necessitates a reflexive consideration of how these factors may have influenced the research process. A female veterinarian and ethicist conducted the majority of the interviews (18 out of 20), while a male ethicist without medical training performed the remaining interviews. This divergence in expertise and gender may have shaped participants’ responses and the dynamics of the interviews, potentially leading to different topics being emphasized based on the interviewers’ perceived authority and identity. To enhance reflexivity, we actively engaged in self-reflection throughout the research process, continuously questioning how our backgrounds, beliefs, and experiences might impact data collection and interpretation. Regular team discussions were held to critically evaluate our approaches and biases, allowing us to gain insights into how our identities influenced the way questions were posed and how responses were understood.

## Conclusions

We conclude that dealing with the conflicting demands of work and private life is of crucial importance for veterinarians. As veterinarians identify strongly with their profession, they run the risk of putting aside their private lives – which is a long-term risk to mental health.

One aspect that should not be overlooked is the fact that our data indicates the following: When veterinarians describe mental load, it is primarily due to the animal owner and their situation, rather than the animal’s situation.

The exchange of experiences and coping strategies between veterinarians should be encouraged in this context, for example, the experience of veterinarians who have been working in the profession for many years could be helpful for newcomers when it comes to the question of successful boundary management.

As our current understanding of veterinarians’ concrete boundary management is still limited, future research tasks are manifold. It would be advisable to test the findings of the present study through quantitative surveys, also with a view to investigate other veterinary fields of work and/or possible differences between self-employed and employed veterinarians. In addition, the permeability of the boundaries “in the other direction” should be examined, i.e., to what extent private life is integrated into professional life and the consequences of this. Finally, given the risks associated with poor mental health, veterinary students should be better prepared for the importance of boundary work in their professional lives.

## Supporting information

S1 File Overview of the participants. (DOCX)

S2 File Interview guideline. (DOCX)

S3 File Code list. (DOCX)
