# Supplementary material for: ‘I’m not just a vet, I’m also a human.’ A qualitative interview study on boundary management between work and private life among small animal veterinarians
Source: PLoS One. 2025 May 9;20(5):e0322938. doi: 10.1371/journal.pone.0322938 (PMC12064008; doi:10.1371/journal.pone.0322938)
Supplement: S2 File — (DOCX) [file pone.0322938.s002.docx]

Supplementary File 2

# Interview guide

| **Introduction to the interview with an opening question**   - You studied veterinary medicine and now work as a veterinarian. Please tell me a little about yourself and your professional career. You can take as much time as you like and tell me everything you can think of about your professional career and stations or what is important to you. | | |
| --- | --- | --- |
| **Part 1a:** **Guide to the reasons for specializing in hospice and palliative care and/or explicitly offering it** | | |
| *Veterinarians not specialized in the field* | You explicitly offer hospice and/or palliative care as a service on your website.   - *What are the reasons for this and how did you come to this prioritization?* - *In general terms, what does it mean to you to provide palliative care and/or to accompany an animal as it dies?* | |
| *Veterinarians specialized in the field of hospice and palliative care (postgraduate training programme)* | You have specialized in the field of hospice and palliative care.   - *What are the reasons why you have specialized in this area?* - *In general terms, what does it mean to you to provide palliative care and/or to accompany an animal as it dies?* | |
| In recent years, veterinary medicine has seen a rapid increase in the use of modern technologies and methods, as well as an increasing specialization of veterinarians. These developments make it possible to care for animals better than ever before and, at best, to cure them.   - *In your opinion, what role does hospice and palliative care play in small animal medicine today?* - *What do you think it takes to be a good veterinarian in the field of hospice and palliative care?* - [Based on your description, it is clear that it is a balancing act to consider the different interests]. *Do you have a specific case that you remember where this worked very well?* - *Can you tell me about a specific case that was particularly challenging for you in the area of palliative care and end-of-life care?* | | |
| **Part 1b: Relationships and characterization of relationships in hospice and palliative care** | | |
| - *How would you respond if someone said: "Palliative care and end-of-life care for animals is nothing more than a service for the pet owner. The animal gets nothing out of it."* - *How would you describe the caregivers and their relationship with their pet who use hospice and palliative care for their pet?* - *How would you describe the relationship between you and the caregivers in this specific area of work?*    - *What aspects are important to you here?* - *Do your relationships with the caregivers change over the course of the care period? If so, to what extent do they change?* | | |
| **Part 2: Communication and time aspects of hospice and palliative care** | | |
| - *What do caregivers want to talk to you about and what channels do you use to communicate with each other?* - *What do you find particularly challenging about communication and why?* - *What role does time play in hospice and palliative care?* - *How does the care of your patients and their caregivers change for you, knowing that your patient's life span is limited to just a few weeks, for example?* - *Does it make a difference to you whether an animal dies naturally - or by euthanasia? If yes, why? If not, why?* | | |
| **Part 3: Infrastructural requirements and care in the home environment** | | |
| - *What is the structure of a typical working day or working week for you?* - *Imagine that: Money doesn't matter. What would you change or wish for so that you could offer your work in the field of hospice and palliative care even better?* | | |
| *Veterinarians working in an office/practice* | | - *Do you also care for animals at home?* - *If so, what advantages and challenges do you face when caring for patients at home?* |
| *Mobile veterinarians* | | You are working as a mobile vet and look after your patients during home visits.   - *What are the advantages and challenges of caring for patients at home?* |
| **Part 4a: Conclusion: Dying and death of animals and its impact on contemporary society** | | |
| - *Has your view of dying and death changed as a result of your work in the field of hospice and palliative care in general? If so, how?* - *Do you believe that caregivers who consciously decide to provide palliative care and end-of-life care for their pet have a different view of dying or death or develop a different view during the process?*   People often experience "dying" and "death" primarily through the loss of their own pet or an animal in their circle of family and friends.   - *In your opinion, what influence does the death of animals have on the way we deal with dying and death in our society in general?* | | |
| **Part 4b: Wishes for the future of small animal medicine** | | |
| If you recall what we have discussed today:   - *What would you wish for the future generation of small animal veterinarians?* - *And what would you wish for from the future generation of small animal veterinarians?* | | |
| **Part V: Conclusion** | | |
| - *Can you think of anything else on the subject of palliative care and end-of-life care for animals in small animal medicine that you have not yet mentioned? Or would you like to add something?*   **Thank you very much for the interview and your time!** | | |
